# Association of rib anomalies and childhood cancers

**DOI:** 10.1038/bjc.2011.366

**Published:** 2011-09-13

**Authors:** H Zierhut, M Murati, T Holm, E Hoggard, L G Spector

**Affiliations:** 1 Departments of Pediatrics and Masonic Cancer Center, University of Minnesota, MMC 484, 420 Delaware Street SE, Minneapolis, MN 55455, USA; 2 Department of Radiology, University of Minnesota Amplatz Children's Hospital, MMC 292, 420 Delaware St SE, Minneapolis, MN 55455, USA; 3 Departments of Pediatrics and Masonic Cancer Center, University of Minnesota, MMC 715, 1-187 Moos, 420 Delaware Street SE, Minneapolis, MN 55455, USA

**Keywords:** childhood cancer, rib anomalies, congenital anomalies

## Abstract

**Background:**

Congenital anomalies have been found more often in children with cancer than in those without. Rib abnormalities (RAs) have been associated with childhood cancer; however, studies have differed in the type of RAs and cancers implicated.

**Methods:**

Rib abnormalities were assessed predominantly by X-ray in a hospital-based case–control study.

**Results:**

There was a significant difference in the number of cases *vs* controls with RAs after controlling for age and sex, specifically for acute myelogenous leukaemia, renal tumours, and hepatoblastoma.

**Conclusion:**

The results of this study support previous reports that there is an association of rib anomalies with childhood cancer.

Multiple studies have demonstrated an association between morphological abnormalities and paediatric cancer (Evans *et al*, 1993; Narod *et al*, 1997; Merks *et al*, 2008). Associations between congenital anomalies and cancer predisposition syndromes are noted in single gene disorders such as Gorlin syndrome, Fanconi anaemia, and Wilm's tumour 1 mutation-related disorder (WT1) (Pelletier *et al*, 1991; Cowan *et al*, 1997; Kimonis *et al*, 1997; Alter *et al*, 2003). Even in the absence of single gene disorders, several epidemiological studies have provided data showing an association between childhood cancer and rib anomalies (RAs; Schumacher *et al*, 1992; Merks *et al*, 2005; Loder *et al*, 2007). Normally, an individual has 12 pairs of ribs with a total of 24 ribs. Abnormalities of the ribs can be numerical (e.g., >24 or <24 ribs) or structural (e.g., cervical ribs, bifid ribs, synostoses, and segmentation defects). All three previous studies examining RAs have reported an association with childhood cancer; however, the studies differed in the specific type of RAs implicated. This study sought to better understand and more robustly describe the association between childhood cancer and morphological defects of the ribs in a US population.

## Materials and methods

### Subject selection

Rib anomalies were assessed in a hospital-based case–control study. Cases consisted of all paediatric haematology and oncology and bone marrow transplantation (BMT) patients treated at the University of Minnesota Medical Center—Fairview in Minneapolis, MN for malignancy during 2003–2009. Cases must have been diagnosed between the ages of 0–19 years and had been imaged during the study period. Children with a known syndrome (e.g., bone marrow failure syndromes, Down's syndrome, and muccopolysaccaridoses) identified through our database were excluded; we expect that only very minimal number of syndromic cases were missed. Controls were randomly selected paediatric patients who received a chest X-ray at Fairview Ridges Hospital in Burnsville, MN during June and October of 2003–2008. Controls were chosen from this community hospital as they more likely represent the general Twin Cities paediatric population. Indications for chest X-rays in controls included asthma or shortness of breath, bronchitis, chest pain, possible pneumonia, trauma, and others (e.g., foreign body). The study was approved by the University of Minnesota Institutional review board.

### Data collection and quality assurance

Images were reviewed for numerical and structural RAs according to previously described scoring methods (Coury and Delaporte, 1954; Merks *et al*, 2005). The latter were cervical ribs (left, right, or bilateral not including transverse apophysomegalies), bifid ribs (left, right, or bilateral), rib synostoses/fusion (left, right, and bilateral), vertebral segmentation anomaly, and post-surgical repairs. Chest X-rays were evaluated whenever possible, with additional images evaluated when clarification was needed. When a chest X-ray was unavailable, magnetic resonance imaging or computed tomography (CT) was evaluated. An electronic database was created using FileMaker Pro 10 software (Filemaker Inc., Santa Clara, CA, USA) to assist in abstracting data. The abstraction instrument was first tested by two radiologists independently reviewed a random sample of 100 images to assess the interrater reliability (*κ* statistic) of the abstraction tool. The initial abstraction tool showed substantial agreement for the availability of images (Kappa (*κ*)=1.00), rib number (*κ*=0.75), and bifid ribs (*κ*=0.79) (Landis and Koch, 1977). Moderate agreement was noted for the evaluability of images (*κ*=0.57) and cervical ribs (*κ*=0.52). Improvements to the abstraction tool included clarification of the definition of which images were considered not evaluable due to the inability to clearly visualise the C7 and L1 vertebra. After the original review of images, two radiologists analysed all positive images. Of the 82 images scored positive by the resident, the radiologists found 77 positive images indicating a 94% agreement. A complete reanalysis of the radiologists’ data found no substantial differences compared with the resident (data not shown).

Using the improved abstraction instrument one radiology resident reviewed each radiograph, followed by *post hoc* evaluation of a random sample of 100 images by two radiologists to determine interrater reliability of availability of images (*κ*=1.0), evaluability of images (*κ*=0.80), and the presence of RA (*κ*=0.85). The high level of agreement between the radiologists and radiology resident provided confidence to allow the resident's evaluations to stand in the analysis. Blinding reviewers to case status was not entirely possible; however, they were not made aware of the study's hypothesis until completion of data collection.

### Statistics

Pearson's *χ*^2^-test was used to assess categorical data differences between cases and controls. Dichotomous variables were created for normal or abnormal ribs (any RA including abnormal rib number, cervical ribs, bifid ribs, and rib synostoses), rib number (24 and <24, >24), cervical and bifid ribs. Only one rib synostoses/fusion or segmentation defects were detected and so these were not analysed. All cases as well as individual cancer types with greater than five RAs were analysed. Unconditional logistic regression was used to calculate the ORs and 95% CI for RAs adjusting for age and sex.

Sensitivity analyses were completed to examine the possibility of bias introduced from the selection of cases and controls; these included analyses with each indication for X-ray in controls dropped in turn, exclusion of all individuals who resided outside the state of Minnesota, restriction by image type, and separate analyses of paediatric BMT and haematology/oncology patients. All statistical analyses were performed using SAS Version 9.2 software (SAS Institute Inc., Cary, NC, USA).

## Results

There were 625 eligible cases (paediatric haematology/oncology, *n*=409, BMT, *n*=216) and 1499 eligible controls (Table 1). Controls had a higher percentage of available images (93.2%), but a lower percentage of evaluable images (81.2%) (Table 2). Cases that had cancer types not frequently requiring chest imaging (e.g., brain tumours) were more likely to have no images available but on the whole most cases had multiple chest X-rays or CT images. Controls on the other hand were selected based on having had a single chest X-ray and the indications for imaging were less likely to necessitate a chest X-ray of the entire rib cage or follow-up imaging. Reasons for non-evaluation included inability to visualise cervical vertebra (C7), lumbar vertebra (L1), both C7 and L1 or poor quality. The resulting ratio of evaluable images for cases to controls was 1 : 2.5. The radiologist used various means for identifying RAs with the preference first via X-ray followed by CT scan. The image types available differed in cases and controls (*P*<0.0001). Controls had a higher percentage of X-rays (99.6% *vs* 88%) and cases had a higher percentage of available CTs (12.0% *vs* 0.2%) (Table 1). Study participants varied significantly by age at first chest imaging, ethnicity, and residence but did not differ by gender (Table 2).

Rib number assessed as an integer differed in cases and controls (Fisher's exact *P*-value=0.008) (Table 3). When categorised as less than or greater than 24 ribs, the crude analysis was borderline significant with an OR of 1.57 (95% CI: 0.98, 2.53) and when adjusted for age and sex attained significance with an OR of 1.66 (95% CI: 1.00, 2.74) (Table 4). A similar association was seen after excluding BMT cases (OR=1.78, 95% CI: 1.01, 3.12).

Presence of any RA was also borderline significant in the crude analysis (OR=1.55, 95% CI: 0.98, 2.46), but significant after adjustment (OR=1.60, 95% CI: 1.0, 2.65) (Table 4). Removing BMT cases strengthened the association (OR=1.81, 95% CI: 1.05, 3.10). Cervical ribs were the only structural RA with sufficient numbers of events to permit analysis. A description of all RAs is available via online Supplementary Table 1. The inclusion of all cases demonstrated no association between cervical ribs and paediatric cancer in the crude and adjusted models. Removal of BMT cases increased the OR, but it was not significant.

Individual cancer types with substantial numbers of RAs (*n*>15) are noted in Table 5. Cases with renal tumours and acute myelogenous leukaemia (AML) had a statistically significant increased odds of RAs compared with all controls in both the crude and adjusted models. Hepatoblastoma was not included in our main analysis due to a limited number of cases, but two out of the five cases had a RA (OR_crude_=14.14, 95% CI: 2.34, 88.83, OR_adjusted_=14.43, 95% CI: 2.34, 88.83). All other cancer types did not show a significant association with RAs (Table 5). The same analysis was completed for the association of abnormal rib number as opposed to total RAs and individual cancer type with similar findings (data not shown).

Dropping each control X-ray indication or BMT cases did not markedly alter results (data not shown). Excluding all individuals living outside the state of Minnesota or X-ray only images greatly reduced the number of analysable cases making the associations essentially null. Interestingly, when RAs and rib number were separated into two ethnic categories (Caucasian and non-Caucasian) a statistically significant result was noted for non-Caucasians (OR=2.13, 95% CI: 1.1, 4.08) but not in Caucasians alone (OR=1.06, 95% CI: 0.44, 2.53).

## Discussion

We detected a moderate but fairly consistent association of RAs and childhood cancer, which remained despite several sensitivity analyses that we performed to compensate for limitations. The findings further appeared to be strongest in AML, renal tumours, and hepatoblastoma. Our results offer partial confirmation of three previous studies on the topic. Loder *et al* (2007) and Schumacher *et al* (1992) reported an association of RAs with abnormal rib number whereas Merks *et al* (2005) failed to confirm this association. Merks *et al* and Schumacher *et al* (1992) found an association of cervical ribs and overall childhood cancer, but the studies showed discrepant results between which childhood cancers were associated with cervical ribs. It should be noted that transverse apophysomegalies were not included in our definition of cervical ribs, which may account for the lack of a positive association found in our study. Our results are similar to Loder *et al* (2007) with a small percentage of identifiable cervical ribs and a significant association of abnormal rib number with paediatric cancer. We did not see significant associations with other cancers previously reported with the exception of Wilm's tumour (Schumacher *et al*, 1992). Our results exhibited an increased number of RAs in CNS neoplasms but the result was not significant. A reduced number of CNS and miscellaneous intracranial and intraspinal neoplasms in our analysis may have prohibited power to detect a significant association.

The US study by Loder *et al* (2007) is closest to our population and may account for the similarity of results between the studies. Data on RAs may not be generalisable to all ethnic populations. To the extent that we could rely on the data supplied and examine ethnicity (Caucasian and non-Caucasian), we found an association limited to non-Caucasians although this is not a large percentage of our study population. Future studies may help to better understand the role of ethnicity on RAs and the association with childhood cancer.

The hospital-based case–control study design had several limitations. The study population was created from a convenience sample which to some extent limited our analyses. Case and control selection has the potential to introduce bias. Cases in our study were obtained from a tertiary care centre. Cases were likely unaware of RAs and therefore unlikely to be differentially ascertained based on this basis. The underlying cohort that gave rise to the cases in our study would be difficult to define. In an attempt to best replicate the cohort, controls were obtained from a hospital-based clinic to better represent the paediatric population of Minnesota, but we cannot rule out bias due to mismatch between sources of cases and controls. Controls were included in our study for a variety of conditions to dilute any effect of one condition increasing the likelihood of obtaining a chest X-ray due to having a RA.

Our results indicate a modest but fairly robust association between RAs and paediatric cancer generally in line with other findings. Particularly, RAs were associated with increased odds of the individual cancer types AML, renal tumours, and hepatoblastoma. Paediatric cancer aetiology remains an elusive area of research with great need for better understanding. Prospective studies examining the genetics of RAs and childhood cancer may provide insight into the pathways leading to the development of paediatric cancer. The existence of common genetic and/or developmental origins in congenital anomalies and childhood cancer is a rich area for future investigation.

## Figures and Tables

**Table 1:** Description of images available and evaluable

**Eligible participants**	**Cases (*n*=625)**	**Controls (*n*=1499)**	***P*-value**
Available images	478 (78.5%)	1398 (93.2%)	
			
*Evaluable images*	459 (96.0%)	1135 (81.2%)	
X-ray	404 (88.0%)	1131 (99.6%)	<0.0001
CT	55 (12.0%)	2 (0.2%)	
Other	0 (0.0%)	2 (0.2%)	
			
*Non-evaluation images*	19 (4.0%)	263 (18.8%)	
Both C7 and L1 not visible	0 (0%)	1 (0.3%)	0.94
C7 not visible	10 (52.6%)	133 (50.5%)	
L1 not visible	9 (47.4%)	125 (47.5%)	
Poor quality	0 (0%)	4 (1.5%)	

Abbreviation: CT=computed tomography.

**Table 2:** Demographics of paediatric cancer cases and controls

	**Cases (*n*=459)**	**Controls (*n*=1135)**	***P*-value**
Age at first chest image (years, s.d.)	9.42 (5.91)	5.57 (5.65)	<0.0001
Age at diagnosis	8.19 (5.91)	—	
			
*Gender* a			
Female	182 (40.3%)	494 (43.6%)	0.23
Male	270 (59.7%)	639 (56.4 %)	
			
*Ethnicity* a			
African-American	7 (3.5%)	111 (12.8%)	<0.001
Asian	9 (4.5%)	30 (3.5%)	
Caucasian	173 (84.6%)	627 (72.7%)	
Hispanic/Latino	7 (3.5%)	50 (5.8%)	
Other	8 (4.2%)	45 (5.2%)	
			
*Residence*			
Local (MN resident)	302 (67.4%)	1108 (97.9%)	<0.0001
Non-local	146 (32.6%)	24 (2.1%)	
			
*Indications for chest X-ray in controls* a			
Asthma or shortness of breath	—	187 (16.5%)	
Bronchitis	—	256 (22.5%)	
Chest pain	—	105 (9.3%)	
Possible pneumonia	—	416 (36.7%)	
Trauma	—	46 (4.0%)	
Other	—	122 (10.8%)	

a Sex was missing for 2 controls and 7 cases, ethnicity is missing for 272 controls and 254 cases, and indications for chest X-ray were missing in 2 controls.

**Table 3:** Rib number in paediatric cancer cases and controls

	**Cases (*N*=455)^a^**	**Controls (*N*=1133)^a^**	***P*-value**
20	0 (0%)	1 (0.1%)	0.008
22	10 (2.2%)	24 (2.1%)	
23	8 (1.8%)	2 (0.2%)	
24	426 (93.6%)	1086 (95.9%)	
25	4 (0.9%)	12 (1.1%)	
26	7 (1.5%)	8 (0.7%)	

a Any noticeable surgical alterations effecting the rib cage were excluded from the analysis (control=2 and cases=4).

**Table 4:** Description of rib anomalies in paediatric cancer cases and controls

	**Total cases (*N*=455)**	**Total controls (*N*=1133)**	**Crude OR^a^ (95% CI), *P*-value**	**Adjusted OR^b^ (95% CI), *P*-value**	**Adjusted OR^b^ without BMT (95% CI), *P*-value**
Any rib anomalies	31 (6.8%)	51 (4.5%)	1.55 (0.98, 2.46), *P*=0.06	1.60 (1.00, 2.65), *P*=0.05	1.81 (1.05, 3.10), *P*=0.03
Rib number (<24 or >24)	29 (6.4%)	47 (4.1%)	1.57 (0.98, 2.53), *P*=0.06	1.66 (1.00,2.74), *P*=0.05	1.78 (1.01, 3.12), *P*=0.05
Cervical ribs	6	9	1.67 (0.60, 4.72), *P*=0.13	1.63 (0.55, 4.80), *P*=0.38	2.61 (0.89, 7.68), *P*=0.08

Abbreviations: OR=odds ratio; 95% CI=95% confidence interval; BMT=bone marrow transplantation.

Any rib anomaly including abnormal rib number, cervical ribs, bifid ribs, and rib synostoses. The total rib anomalies are not the sum of abnormal rib number and rib abnormalities due to some individuals who had both abnormal rib number and abnormality. Each case was only counted once. Any noticeable surgical alterations were excluded from the analysis (control=2 and cases=4).

a Fisher's exact test was used to determine the *P*-value for cervical ribs in the crude analysis.

b Adjusted for sex and age at first chest imaging.

**Table 5:** Rib anomalies identified by individual cancer types

		**Normal ribs**	**Abnormal ribs**	**Crude OR (95% CI)**	**Adjusted OR (95% CI)**
Total cancers	455	424 (93.2%)	31 (6.8%)	1.55 (0.98, 2.46)	1.60 (1.0, 2.65)
					
*Leukaemias, myeloproliferative diseases, and myelodysplastic*	221	206 (93.2%)	15 (6.8%)	1.54 (0.85, 2.80)	1.55 (0.84, 2.88)
Lymphoid leukaemias	105	99 (94.3%)	6 (5.7%)	1.40 (0.58, 3.36)	1.27 (0.51, 3.11)
Acute myeloid leukaemias	78	70 (89.7%)	8 (10.3%)	2.42 (1.11, 5.31)	2.29 (1.02, 5.13)
Chronic myeloproliferative diseases	20	20 (100%)	0 (0.0%)	—	—
Other specified or unspecified leukaemias	18	17 (94.4%)	1 (5.6%)	1.36 (0.18, 10.44)	1.16 (0.15, 8.98)
					
*Lymphomas and reticuloendothelial neoplasms*	50	49 (98.0%)	1 (2.0%)	0.47 (0.06, 3.49)	0.37 (0.05, 2.89)
Hodgkin lymphomas	29	29 (100%)	0 (0%)	—	—
Non-Hodgkin lymphomas (except Burkitt's lymphoma)	12	11 (91.7%)	1 (8.3%)	2.10 (0.27, 16.63)	1.67 (0.21,13.41)
Other specified or unspecified lymphomas	9	9 (100%)	0 (0%)	—	—
CNS and miscellaneous intracranial and intraspinal neoplasms	34	31 (91.2)	3 (8.8%)	2.24 (0.66, 7.59)	2.00 (0.59, 6.77)
Neuroblastoma and other peripheral nervous cell tumours	31	29 (93.6%)	2 (6.4%)	1.46 (0.34, 6.30)	1.48 (0.34, 6.40)
Renal tumours	20	17 (85.0%)	3 (15.0%)	3.74 (1.06, 13.19)	3.73 (1.05, 13.22)
					
*Malignant bone tumours*	39	37 (94.9%)	2 (5.1%)	1.15 (0.27, 4.89)	0.95 (0.21, 4.31)
Osteosarcomas	21	19 (90.4%)	2 (9.5%)	2.23 (0.51, 9.85)	1.81 (0.38, 8.53)
Ewing tumour and related sarcomas of bone	15	15 (100%)	0 (0%)	—	—
Other specified and unspecified malignant bone tumours	3	3 (100%)	0 (0%)	—	—
					
*Soft tissue and other extraosseous sarcomas*	30	29 (96.7%)	1 (3.3%)	0.73 (0.10, 5.41)	0.64 (0.08, 4.91)
Rhabdomyosarcomas	15	14 (93.3%)	1 (6.7%)	1.52 (0.20, 11.75)	1.35 (0.17, 10.62)
Other specified or unspecified soft tissue sarcomas	15	15 (100%)	0 (0%)	—	—
Controls	1133	1082 (95.5%)	51 (4.5%)	—	—

Abbreviations: OR=odds ratio; 95% CI=95% confidence interval; CNS=central nervous system.
