# Supplementary material for: Association of rib anomalies and childhood cancers
Source: Br J Cancer. 2011 Sep 13;105(9):1392–5. doi: 10.1038/bjc.2011.366 (PMC3241545; doi:10.1038/bjc.2011.366)
Supplement: Supplementary Table 1 [file bjc2011366x1.doc]

On-line Supplemental

Table 1.

| **Rib anomaly** | **Cases (n=31)** | **Controls (n=51)** |
| --- | --- | --- |
| Aplasia 12^th^ ribs | 10 | 24 |
| Cervical ribs |  |  |
| - Bilateral | 5 | 6 |
| - Right | 1 | 3 |
| - Left | 0 | 0 |
| Lumbar ribs |  |  |
| - Bilateral | 2 | 2 |
| Bifurcation |  |  |
| - Right | 0 | 2 |
| - Left | 0 | 2 |
| Synostosis | 1 | 0 |
| Abnormal rib number |  |  |
| - 20 | 0 | 1 |
| - 23 | 8 | 2 |
| - 25 | 3 | 9 |
| Other (N/S) | 1 | 0 |
